# Influence of Silane Sol Sealing Treatment on the Anti-Corrosion of Micro-Arc Oxidation Coating

**DOI:** 10.3390/molecules31071214

**Published:** 2026-04-07

**Authors:** Wei Song, Yasheng Xing, Xueli Xu, Huanxin Li, Weifeng Li, Peng Zhang, Yizhan Li

**Affiliations:** 1 School of Biological and Chemical Engineering, Nanyang Institute of Technology, No. 80 Changjiang Road, Nanyang 473004, China; 2 Henan Key Laboratory of Industrial Microbial Resources and Fermentation Technology, Nanyang Institute of Technology, Nanyang 473004, China; 3 Nanyang Branch of Henan Boiler and Pressure Vessel Inspection Technology Research Institute, No. 1088 Gongye South Road, Nanyang 473000, China; 4 Henan Xibao Metallurgical Group Co., Ltd., Nanyang 474550, China

**Keywords:** aluminium alloy, anti-corrosion, sealing treatment, micro-arc oxidation, silane sol

## Abstract

Silane sol was applied to seal the pores in a micro-arc oxidation coating, with the results proving that the treatment increased the anti-corrosion characteristics of aluminium alloy. Moreover, an electrochemical workstation was employed to test the open-circuit voltage, polarisation potential, and polarisation current of the samples. According to the results, after the aluminium alloy was treated with the micro-arc oxidation coating and underwent subsequent sealing treatment, the open-circuit potential increased from −0.64 to −0.44 V, the corrosion potential from −0.54 to −0.31 V, and the corrosion current density from 56.23 × 10^−7^ to 7.76 × 10^−7^ A. However, when samples were corroded by 1 mol/L HCl, the corrosion potential and corrosion current density decreased to −0.34 V and 20.42 × 10^−7^ A, respectively, proving that sealing the pores on the micro-arc oxidation coating only prevented substrate corrosion for a short time. In addition, slow-strain-rate stretching experiments were conducted to explore the mechanical performances of the samples, determining that the surface treatment had an insignificant effect on the stress of the aluminium alloy but had an important effect on its elongation, and when the surface of the alloy was treated with micro-arc oxidation coating, its elongation decreased from 28% to 26%.

## 1. Introduction

With the increasing depletion of global energy resources, the continuous exploration for oil and gas resources to meet the energy demand has received growing attention [1]. Since shallow oil and gas resources have already been explored and exploited, deep-subsurface and deep-sea resources have become the main focus of petroleum exploration and exploitation to meet the energy needs of industrial activity [2]. As the drilling depth increases, the formation temperature and the abrasiveness of the rock also increase, posing new challenges for the performance of drill pipe materials. Meanwhile, the increase in the total weight of the drill pipe also places severe demands on the hook load of the drilling rig [3]. Therefore, due to their high density and propensity for hydrogen embrittlement during the exploitation of acidic gas fields, steel drill pipes can no longer meet the requirements of deep-well drilling. Aluminium alloy exhibits the properties of low density, non-magnetism, high specific strength, and easy processing; therefore, it is an ideal material to replace traditional steel drill pipes in ultra-deep drilling and extended-reach wells [4].

However, during the petroleum extraction process, to enhance the recovery rate of oil and gas, it is necessary to continuously inject brine into the oil wells. As a highly reactive metal, aluminium is likely to undergo oxidation and corrosion in a brine environment, after which a dense oxide coating can be generated on the metal surface, preventing further corrosion inside the metal [5]; however, this oxide coating can be destroyed in both acidic and alkaline solutions, leading to severe pitting corrosion [6]. Compared with pure aluminium, the corrosion resistance of aluminium alloys deteriorates even more because they contain other alloying elements. When aluminium alloys are used as structural components, they are simultaneously subjected to external forces and the action of corrosive environmental atmospheres; consequently, under the combined action of stress and corrosive media, the pitting corrosion of aluminium alloys is more likely to occur.

Surface treatment is normally used for improving the corrosion resistance of materials [7]. Although various surface treatment methods such as coating, physical vapour deposition [8], and chemical conversion [9] can be adopted to treat the aluminium alloy surface, the coating method attains insufficient coating–substrate bonding force, the physical vapour deposition method is costly, and the chemical conversion method results in an insufficient coating thickness, making it difficult to effectively provide corrosion protection for the matrix of aluminium alloy components. The surface treatment method of preparing and growing an aluminium oxide coating on the aluminium alloy surface by electrochemical means can enhance the corrosion resistance of the alloy components and has become a common method for aluminium alloy surface treatment [10,11,12,13].

At present, there are two main methods for preparing the aluminium oxide coating on the aluminium alloy surface by electrochemical means [14]: One is to use aluminium alloy as the anode and prepare an anodized coating at a relatively low voltage in acidic electrolytes such as sulfuric acid [15], phosphoric acid [16], and organic acids [17]. Since the coating preparation and growth process occur while the material is being dissolved, and growth occurs simultaneously in the acidic electrolyte, the coating is full of through-holes and can only be used for short-term anti-corrosion effects after being sealed [18]. In an alkaline electrolyte with aluminium alloy as the anode, the oxygen plasma discharge of the electrolyte on the surface of the aluminium alloy can be achieved under a relatively high operating voltage [19]. The generated high temperature promotes the interaction between fresh oxygen plasma and aluminium ions, thereby contributing to the preparation and growth of a crystalline aluminium oxide coating on the aluminium alloy surface [20]. The main reaction equations are as follows:

Negative electrode: 2 H_2_O + 2 e = H_2_ + 4HO^−^ (1)

Positive electrode: Al^3+^ + 3 OH^−^ = Al(OH)_3_ − 77.1 kJ/mol (2)

2 Al(OH)_3_ = Al_2_O_3_ + 3 H_2_O + 194.6 kJ/mol (3)

4 OH^−^ = 2 H_2_O + O_2_ + 4 e − 46.6 kJ/mol (4)

Accompanied by the growth of the crystalline aluminium oxide coating, the micro-arc discharge phenomenon continuously occurs on the aluminium alloy surface. In this regard, the aluminium oxide coating prepared by this method is called the micro-arc oxidation (MAO) coating. Given that the MAO coating is mainly composed of crystalline aluminium oxide, it achieves significantly superior corrosion resistance compared to that of the anodized coating [21]. In the preparation of the MAO coating, the aluminium oxide coating is repeatedly broken down with the continuous increase in the oxygen plasma discharge voltage, and the fresh oxygen plasma is under continuous crystalline oxidation with aluminium ions at high temperatures, thus increasing the coating thickness [22]. Consequently, the thicker coating is associated with the higher external voltage required to break it down, and the higher discharge voltage is linked to the greater oxygen plasma discharge intensity and the increased oxygen release. After the aluminium oxide coating is formed, if the oxygen generated by the self-combination of oxygen plasma cannot escape from the coating in time, the coating will be filled with closed or connected pores [23]. Therefore, when the coating thickness is low, an aluminium oxide coating with a high density can be obtained; however, when the coating thickness exceeds 35 μm, its surface is covered with microholes and microcracks resulting from spark discharge, and it is difficult to obtain an aluminium oxide coating with a high density [24].

Pitting corrosion is typical for aluminium alloys. As a result, when the MAO coating contains many micropores and microcracks, it is difficult to effectively inhibit the corrosion behaviour of aluminium alloys. Only through reducing the quantity and size of the micropores and microcracks in the coating can the density and anti-corrosion effects of the MAO coating be improved. Notably, sealing treatment can efficiently decrease the microhole and microcrack size and quantity in the MAO coating [25,26]. Currently, the MAO coating sealing methods mainly involve the individual or combined use of Ce salt [27], organic silicon [28], polymer organic compounds [29], and nanoparticles [30]. According to the relevant literature reports [31], after sealing treatment, the micropore quantity inside the coating is significantly reduced; moreover, the further development and diffusion of microcracks in the coating are inhibited, thus improving the anti-corrosion effects of the coating.

When Ce salt is used as the sealing agent, it deposits in the pores during its hydrolysis process [27]. Using nanoparticles as the sealing agent, the pores are filled by the adsorption and precipitation of nanoparticles due to an electric field force or chemical bonding [29]. Organic compounds are mostly macro-molecular substances that struggle to enter the pores in the aluminium oxide coating [31]. The MAO coating is mainly composed of inorganic aluminium oxide with hydroxyl groups; if organic silicons such as methylsilane and ethylsilane are used as the sealing agent, they can generate a hydrolysis condensation reaction, filling the pores in the MAO coating while forming an organosilicon coating, which can protect the substrate, improving the hydrophobicity of the coating and better enhancing its corrosion resistance [32]. Based on this theory, many researchers have reported the use of silane sol to seal the pores of an MAO coating [33].

Therefore, to broaden the application of aluminium alloys, an MAO coating was synthesised on an aluminium alloy surface, the pores were sealed with organosilicon, and the anti-corrosion as well as mechanical properties of the samples after corrosion were studied.

## 2. Experiment

### 2.1. Materials and Reagents

All experimental reagents were offered by Tianjin Kemiou Chemical Reagent Co., Ltd. (Tianjin, China). The commercial aluminium alloy 2024 (20 × 20 × 1 mm^3^) served as the specimen, and the components of the alloy are shown in Table 1.

### 2.2. Experimental Process

In brief, two 300 × 300 mm^2^ AISI 321 stainless steel sheets were applied as the cathode. The MAO process was performed within the stirred electrolyte containing 15 g/L Na_2_SiO_3_, 5 g/L KOH, and 5 g/L (NaPO_3_)_6_. To be specific, the sample MAO process was carried out at 20 °C for 15 min in the direct current (DC) pulse supply at a 10 kHz frequency, 0.5 A/cm^2^ constant current density, and 15% duty cycle. When the first micro-arc could be seen, the initial voltage was about 300 V. A cooling cycle system was utilised to maintain the electrolyte temperature. To enhance the anti-corrosion effect on the substrate induced by the coating, silane sol was used to seal the pores in an MAO coating. The silane sol was prepared as follows: tetraethoxysilane and 3-(2,3-epoxy propoxide) propyl trimethoxysilane (KH 560) were mixed into a solution composed of 0.5 mol n-butyl alcohol, 0.1 mol butyl ethylene glycol ester, 0.3 mol butyl ester, and 0.4 mol water. The water/silicon ratio was controlled to be equal to 4:5, and HCl was used to adjust the pH value to 2. Afterwards, the solution was magnetically stirred for 2 h at 60 °C to prepare the sol. After the samples were soaked in the sol for 10 min, they were dried at 40 °C. Since aluminium alloys are more prone to pitting corrosion in HCl solution, a 1 mol/L HCl solution was selected as the corrosion agent to reveal their influence on the anti-corrosion effects of the MAO and sealing treatment, so as to fully study the influence of the combined action of stress and corrosion medium on the substrate [34].

### 2.3. Characterisation

The X-ray diffractometer (XRD, D/max-rB, RICOH, Tokyo, Japan) with a CuKa source was used to examine the phase compositions of different coatings, with a current of 30 mA and an accelerating voltage of 40 kV. Additionally, scanning electron microscopy (SEM, S-4700, Hitachi, Tokyo, Japan) was carried out to explore the surface microstructures of coatings. Simultaneously, the equipped energy-dispersive spectrometer was applied to quantitatively and qualitatively analyse element points, lines, and surfaces. Moreover, the open-circuit potential, Tafel curve, and electrochemical impedance of coatings were determined using an electrochemical workstation (Parstat 4000, Princeton Applied Research, Berwyn, PA, USA). Three-electrode systems were applied with aluminium alloy and the MAO coating, the latter of which was applied after sealing the pores. The primary research goal of this article is to examine the corrosion resistance of MAO coatings subjected to the simultaneous effects of corrosion and stress. Therefore, a strong acid solution of 1 mol/L HCl was chosen as the corrosive agent. Because the MAO coating is a double-layer porous coating, it needs to be soaked in the electrolyte for about 2 h before electrochemical testing. In order to prevent the coating from falling off, a neutral salt solution was chosen to analyse its electrochemical performance. The coating after corrosion in 1 mol/L HCl for 1 h was the working electrode, a platinum sheet was the counter electrode, and saturated calomel was the reference electrode. To determine the corrosion potential and the corrosion current density clearly, the solution of 3.5 wt% sodium chloride was selected. In addition, the sample solution contact area was 1 cm^2^, and the starting and ending frequencies were 100 kHz and 0.01 Hz [35] at a scan rate of 2 mV/s [36], respectively. Following testing, coating data were acquired with Zsimp Win software 3.60. Due to aluminium alloys being more prone to pitting corrosion in HCl solutions, to study the influence of the corrosion performance of the samples in the corrode environment, after the samples were coated and corroded in 1 mol/L HCl at room temperature, the slow-strain-rate stretching experiments were carried out to test the stress corrosion of the samples. The temperature was controlled at ambient temperature, 200 °C, and 400 °C at a 2 mm/min stretching rate.

## 3. Results and Discussion

### 3.1. Microsurface of the Coating

**Figure 1:**
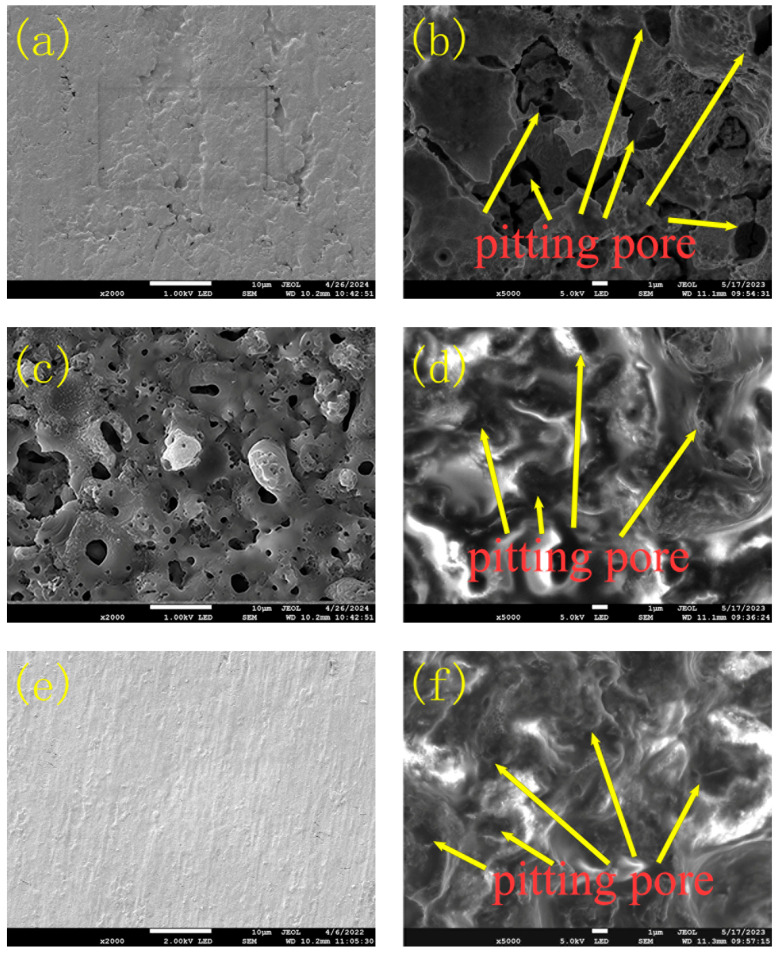
Microsurface of the Al substrate before (**a**) and after (**b**) stress corrosion; MAO coating before (**c**) and after (**d**) stress corrosion; and MAO coating sealed by silane sol before (**e**) and after (**f**) stress corrosion.

The microsurfaces of the samples were investigated through SEM, with the results presented in Figure 1. From Figure 1b, obvious pitting corrosion can be observed on the surface of the aluminium alloy. In addition, based on Figure 1c, when the aluminium alloy was treated with the MAO process, there were micropores all over the surface; after corrosion in the 1 mol/L HCl at room temperature for 1 h, the spot corrosion groove on the sample became shallow (Figure 1d). In Figure 1e, it is discovered that when the MAO coating was sealed by silane sol, its surface was smooth, and pores were not seen in the coating. Figure 1f shows that after the sample was corroded by 1 mol/L HCl, the size of the spot corrosion groove decreased. These results suggest that the MAO coating improved the anti-corrosion effects of the aluminium alloy. In addition, when the coating was sealed with silane sol, it had an enhanced anti-corrosion ability.

**Figure 2:**
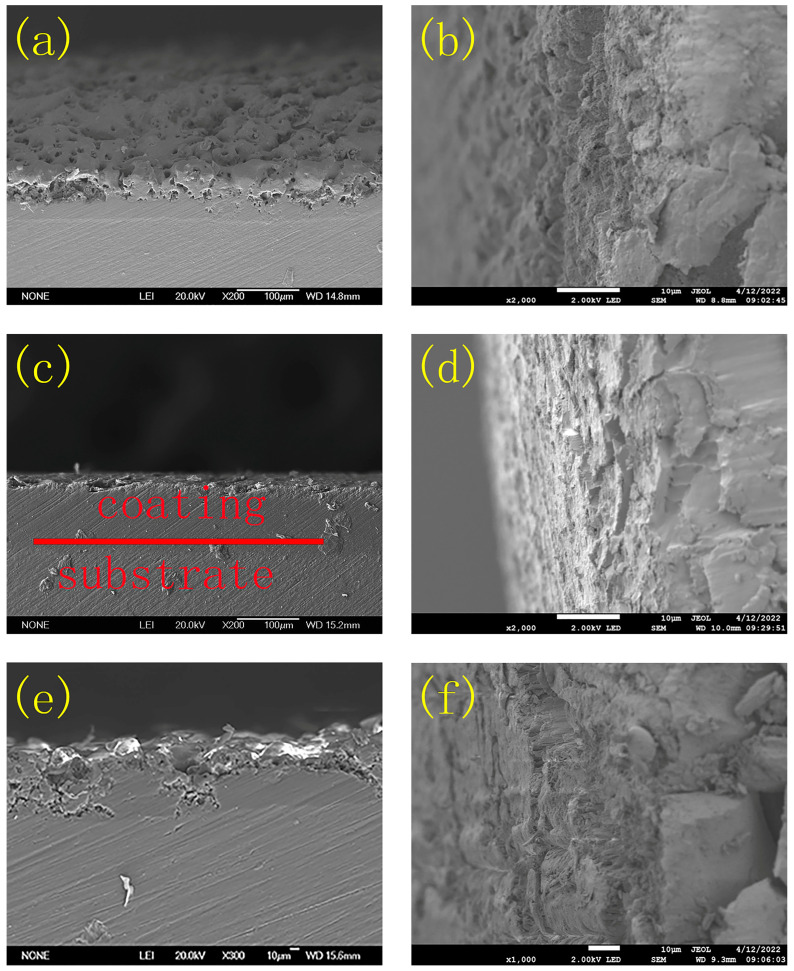
Cross-section of the MAO coating (**a**) before and (**b**) after the stress corrosion; the sealing treatment coating (**c**) before and (**d**) after the stress corrosion; and the corroded coating (**e**) before and (**f**) after the stress corrosion.

The cross-sections of the samples before and after the slow-strain-rate stretching experiments are shown in Figure 2. There were compact and porous layers in the MAO coating, and when the coating was sealed, it was filled, and an obvious porous layer could not be seen in the coating; however, when the coating was corroded, microcracks appeared in the coating. When the samples underwent the slow-strain-rate stretching experiments, the MAO coating with the sealing treatment exhibited higher toughness.

### 3.2. XRD Spectra of the Coating

**Figure 3:**
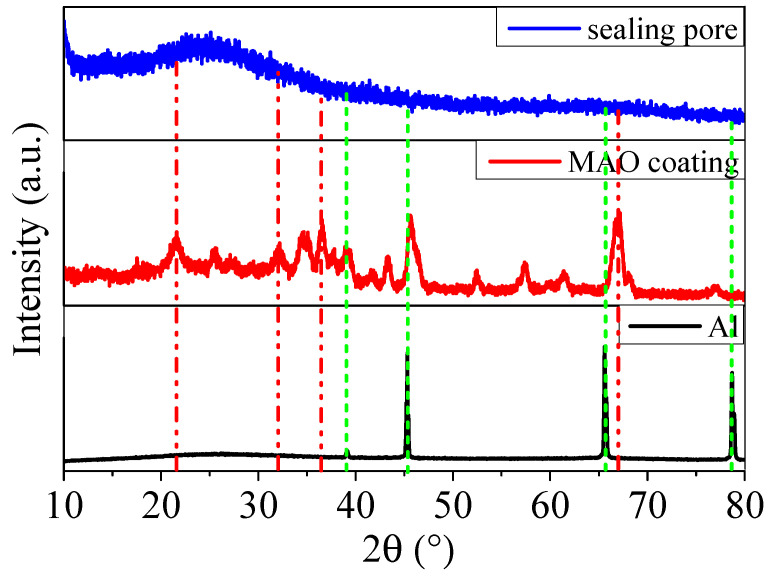
XRD spectra of the aluminium alloy and MAO coating before and after sealing the pores.

We studied the XRD spectra for the aluminium alloy with MAO coating before and after sealing by organic silicon coating, and the results are displayed in Figure 3. From the XRD spectra, there were strong characteristic peaks at 2θ of 45°, 66°, and 79°, consistent with the characteristic peaks of the aluminium alloy [37]. When the aluminium alloy was treated with the MAO process, the characteristic peaks were detected at 2θ of 22°, 32°, 36°, and 67°, in line with the characteristic peaks of [38]. After the coating was sealed by silane sol, there was no obvious characteristic peak observed. These results demonstrated that a crystalline aluminium oxidation coating was synthesised on the aluminium alloy surface by the MAO method, and amorphous silane sol covered the MAO coating surface.

### 3.3. Electrochemical Performance of the Coating

**Figure 4:**
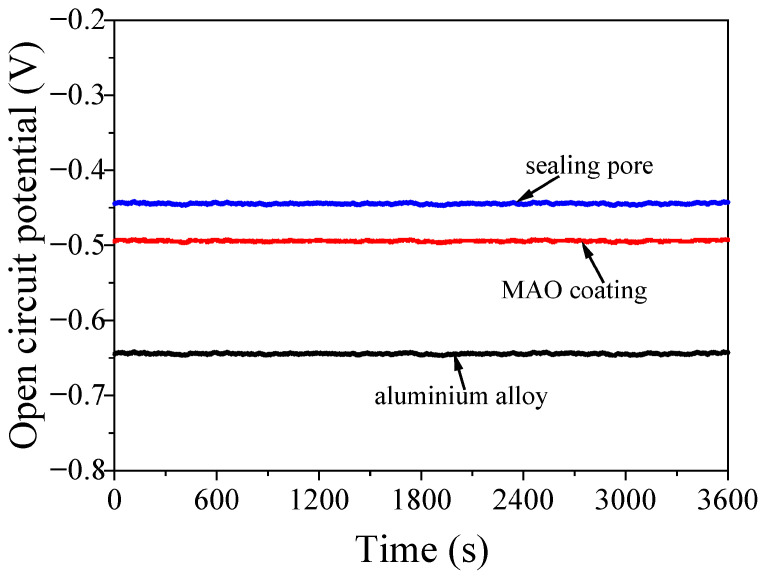
Open-circuit potential of the aluminium alloy and MAO coating before and after sealing the pores at 3.5% NaCl.

Figure 4 shows the open-circuit potential of the coating analysed by the electrochemical workstation. As observed, the open-circuit potential of the aluminium alloy was −0.64 V. When the MAO coating was formed on the aluminium alloy surface, the open-circuit potential increased to −0.49 V, while the value increased to −0.44 V after pores inside the coating were sealed by silane sol. Additionally, the anti-corrosion performance of the sample was enhanced with its increasing open-circuit potential. Based on these findings, the anti-corrosion performance of aluminium alloy was improved after the MAO process and sealing treatment [39].

**Figure 5:**
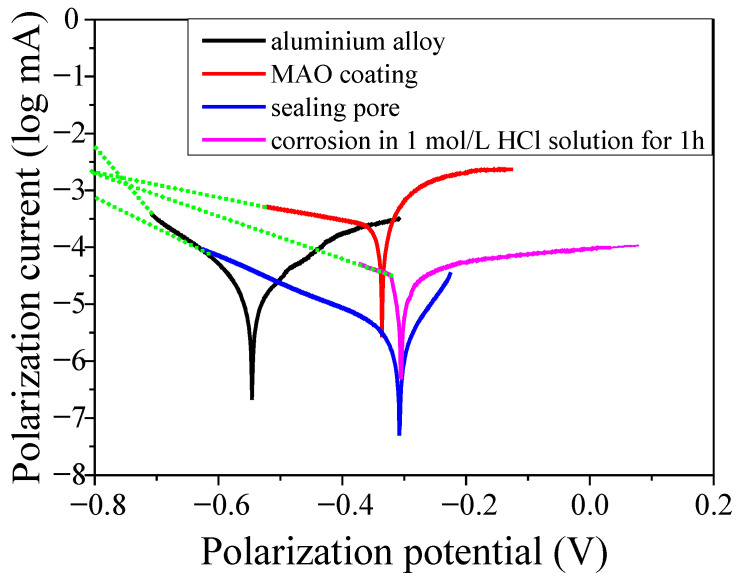
Tafel curve of the aluminium alloy and MAO coating before and after sealing the pores.

Figure 5 and Table 2 present the Tafel curve of the samples. Clearly, the corrosion potential of the aluminium alloy was −0.54 V; after the MAO coating was prepared, the corrosion potential elevated to −0.34 V, and when the MAO coating was sealed by silane sol, it further elevated to −0.31 V. The corrosion potential is an important parameter that reflects whether the material is susceptible to corrosion, with a more negative corrosion potential indicating that the material is more prone to corrosion. Based on our findings, the MAO process and subsequent sealing treatment remarkably improved the anti-corrosion performance of the aluminium alloy. Meanwhile, when the aluminium alloy sample with an MAO coating and sealing performed by silane sol was corroded by HCl solution, the corrosion potential decreased to −0.34 V, proving that in a corrosive environment, the bonding strength between the silane and MAO coating was lower than that of the MAO coating and substrate; thus, the silane coating was peeled off from the MAO coating, and the corrosion potential decreased to −0.34 V [40]. It can also be seen that the corrosion current density of the MAO coating was 21.38 × 10^−7^ A, lower than that of the substrate. When the coating was sealed with silicon sol, the corrosion current density decreased to 7.76 × 10^−7^ A. In contrast, when the sample was corroded in 1 mol/L HCl solution for 1 h, the corrosion current density increased to 20.42 × 10^−7^ A. Such results are associated with the existence of numerous microcracks and micropores in the MAO coating. In addition, pitting corrosion is the typical corrosion behaviour of aluminium alloy. When the aluminium alloy with an MAO coating was corroded in a strong acid solution, the corrosion current density increased; however, when the MAO coating was sealed by silane sol, the corrosion current density decreased because the microcracks and micropores were sealed. Nonetheless, if the silane sol barrier layer was destroyed, the corrosion current density increased. These results prove that a silane sol sealing treatment could improve the anti-corrosion effects of the MAO coating.

**Figure 6:**
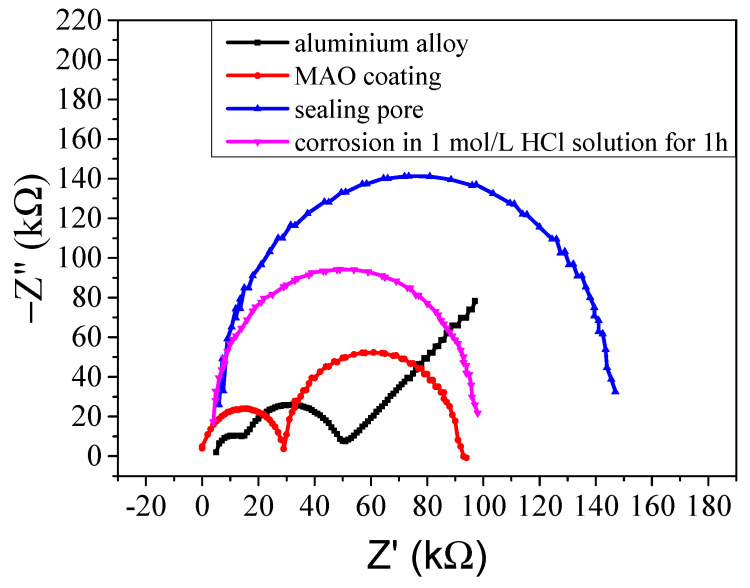
Nyquist curve of the aluminium alloy, MAO coating before and after sealing the pores, and the coating corroded in 1 mol/L HCl for 1 h.

Figure 6 shows the Nyquist curve of the samples. As observed, the impedance of the aluminium alloy was about 45 kΩ, while that of the MAO coating was about 90 kΩ and that after silane sol sealing was about 150 kΩ. There are two semicircles in the aluminium alloy and MAO coating Nyquist curve, indicating that their impedance consisted of the aluminium alloy matrix and their oxide coating on the surface. After the MAO coating sealed the pores, the Nyquist curve became one semicircle because the impedance mainly consisted of the silane sol coating. Similarly to the results shown in Figure 5, when the sample was corroded in the HCl solution, the impedance decreased significantly. As impedance reflects the anti-corrosion performance of the sample [41], the increasing sample impedance demonstrated that the MAO coating and subsequent sealing treatment improved the anti-corrosion abilities of the aluminium alloy.

**Figure 7:**
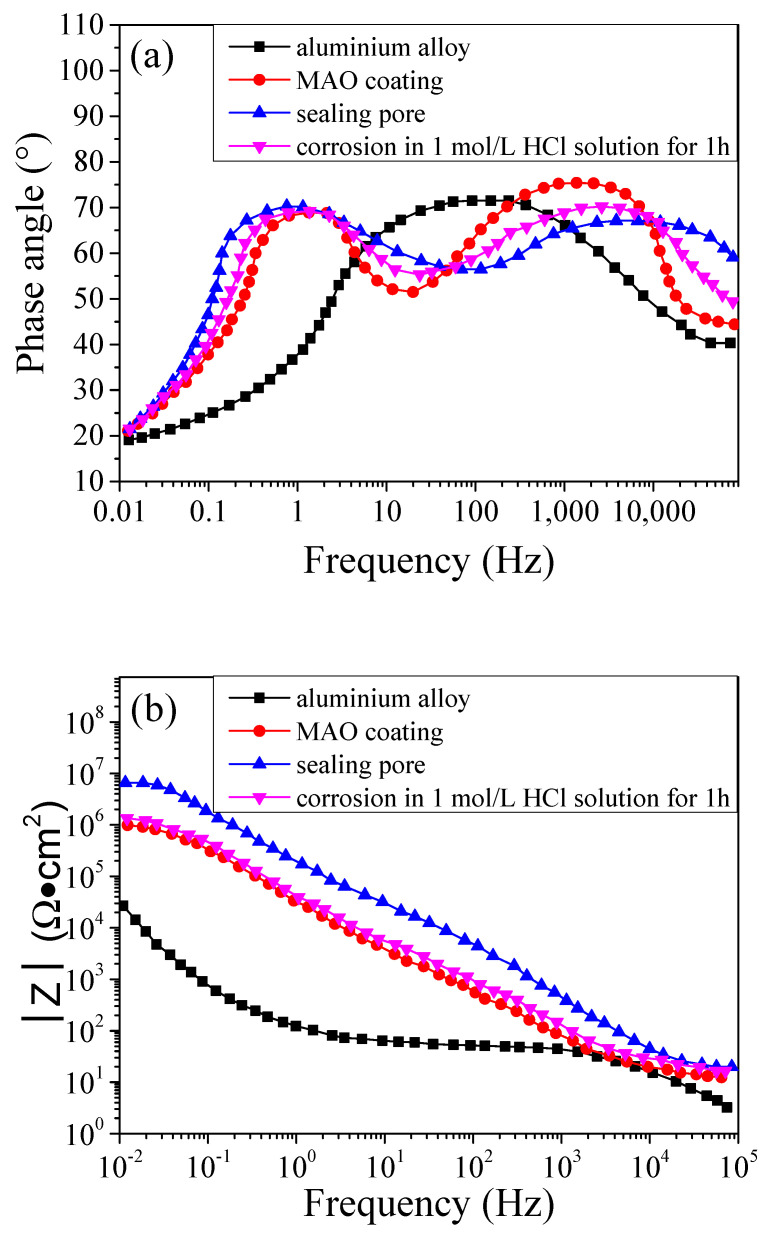
The relationship between the frequency and the (**a**) phase angle and (**b**) modulus of impedance.

We further analysed the electrochemical properties of the coating, the relationship between frequency and phase angle, and the relationship between frequency and modulus of impedance in the samples; the results are shown in Figure 7. It can be seen from Figure 7a that there were extreme peaks when the sample was the aluminium alloy, while there were two extreme peaks when the aluminium alloys were coated. The results indicated that the surface impedance curve of the aluminium alloy contained one time constant, while when it was coated, this increased to two. The two time constants corresponded to the two different coating structures: the porous and the compact coatings. It can be seen from Figure 7b that when the aluminium alloy was coated by MAO, the modulus of impedance increased; after the MAO coating was sealing-treated, the modulus of impedance further increased, but when it was corroded, the modulus of impedance decreased.

**Figure 8:**
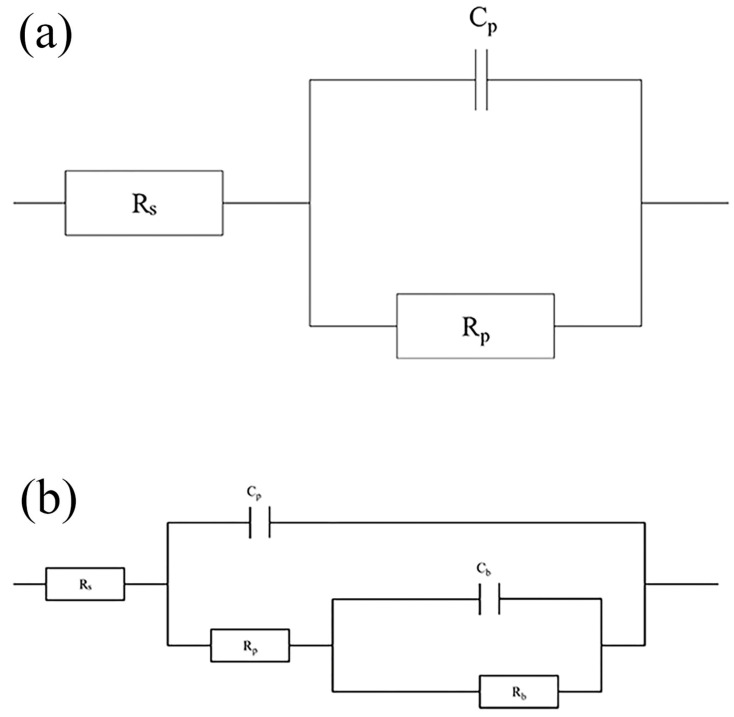
Electrochemical impedance equivalent circuit diagram of (**a**) aluminium alloy, (**b**) MAO coating, MAO coating after sealing the pores, and pore-sealing coating after corrosion in 1 mol/L HCl for 1 h.

Based on a previous analysis of the double-layer structure of the MAO coating and the existing literature [42], the equivalent circuit diagram shown in Figure 8 can perfectly fit the measured electrochemical impedance data. R_s_ represents the series resistance of the electrolyte between the working and reference electrode, R_p_ represents the resistance of the porous layer, R_b_ represents the resistance of the compact layer, C_p_ represents the capacitance of the porous layer, and C_b_ represents the capacitance of the compact layer. n_p_ and n_b_ represent the residual for the fits of C_p_ and C_b_. It can be seen in Figure 8a that the impedance of the aluminium alloy consisted of two parts, and the total impedance of the treated samples consisted of three parts. The first was the solid–liquid interface resistance (R_s_), the second the loose layer resistance. Moreover, the impedance of the MAO coating, the MAO coating after sealing the pores, and the pore-sealing coating after corrosion in 1 mol/L HCl for 1 h consisted of three parts. Compared to the aluminium alloy, there is an additional dense layer. The resistance of the loose layer consisted of impedance (R_p_) and capacitance (C_p_), and that of the dense layer consisted of impedance (R_b_) and capacitance (C_b_). The numerical date table of the equivalent circuit is illustrated in Table 3, showing that the R_s_, C_p_, and C_b_ were low [43], with all of them being lower than 1 Ω. The impedances of the samples were determined by R_b_ and R_p_. When the aluminium alloys were treated by MAO and the pores were subsequently sealed, R_p_ increased from 245.1 to 189,200 Ω and R_b_ from 36,130 to 364,300 Ω. It can also be seen from Table 3 that R_b_ was higher than R_p_, with R_b_ representing the compact layer resistance and R_p_ the porous layer resistance; the results prove that an increase in the resistance of the compact layer could increase the anti-corrosion ability of the coating significantly. However, when the coating was corroded, R_b_ decreased to 206,500 Ω and R_p_ decreased to 54,100 Ω, showing that the anti-corrosion ability of the coating decreased.

### 3.4. Slow-Strain-Rate Stretching Experiments

**Figure 9:**
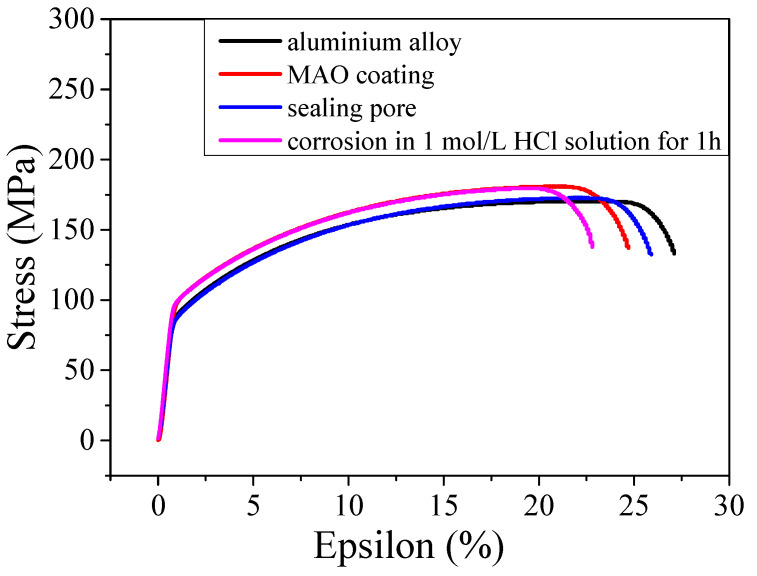
Slow-strain-rate stretching curve of the aluminium alloy, MAO coating, MAO coating after sealing the pores, and MAO coating corroded in 1 mol/L HCl for 1 h.

Pitting is the typical form of corrosion in aluminium alloy and causes the concentration of material forces, thus affecting the material’s mechanical performance. To explore the impact of corrosion on the mechanical performance of aluminium alloy, slow-strain-rate stretching experiments were performed at 200 °C for the aluminium alloy substrate, MAO coating, MAO coating sealed by silane sol, and MAO coating sealed by silane sol after the samples were corroded by 1 mol/L HCl. The results are presented in Figure 9. The tensile strengths of the aluminium alloy substrate, the MAO coating, and the MAO coating with subsequent sealing treatment were not apparently changed, with small changes ranging from 168 to 174 MPa; however, the elongation rate of the material decreased significantly. To be specific, the elongation of the aluminium alloy was 28%, and the value decreased to 25% after MAO treatment. After the pores were sealed, the elongation slightly increased to 26%, while after the material was corroded by the HCl solution, it decreased to 23.5%. These results indicate that the MAO coating and subsequent pore-sealing process decreased the plastic properties of the aluminium alloy. In addition, the corrosion of the aluminium alloy also seriously affected its plasticity. This is mainly because the MAO coating is an aluminium oxide coating with high hardness and high brittleness. When it was prepared on the substrate surface, it weakened the plasticity of the aluminium alloy matrix; however, silane sol was used during the hole-sealing treatment, which reduced the brittleness of the MAO coating to some extent, thereby increasing the elongation rate of the sample. In addition, when the sample was corroded, pitting corrosion occurred, resulting in the stress concentration of the sample and finally a decrease in the elongation rate.

## 4. Conclusions

(1) To increase the anti-corrosion properties of the aluminium alloy, silane sol was used to seal pores in an MAO coating. According to our XRD and SEM results, this sealing was successful.

(2) The electrochemical workstation analysis results showed that after the coating was synthesised on the aluminium alloy surface, the open-circuit potential elevated from −0.64 to −0.44 V, the corrosion potential elevated from −0.54 to −0.31 V, the corrosion current density declined from 56.23 × 10^−7^ to 7.76 × 10^−7^ A, and the impedance increased from 45 to 150 kΩ. However, the slow-strain-rate stretching experiment results revealed that the elongation of the sample decreased when an MAO coating was prepared on its surface. When the pores in the coating were sealed by silane sol, the elongation of the substrate decreased from 28% to 26%. In addition, when the samples were corroded by 1 mol/L HCl, the anti-corrosion performance and elongation rate decreased to 23.5%.

(3) While sealing treatments like silane coating significantly enhanced the anti-corrosion abilities of MAO coatings, the bonding strength between the silane and MAO coating was lower than that of the MAO coating and substrate; thus, the treated coatings would still be corroded when exposed to high-corrosion environments for a long period of time.

## Figures and Tables

**Table 1:** Components of the 2024 aluminium alloy.

Composition	Cu	Si	Fe	Mn	Mg	Cr	Zn	Ti	Al
Wt. %	3.8~4.9	0.5	0.5	0.3~1.0	1.2~1.8	0.10	0.25	0.15	Balance

**Table 2:** Corrosion potential and corrosion current density of the samples.

Sample	Corrosion Potential (V)	Corrosion Current Density (I × 10^−7^ A)
Aluminium alloy	−0.54	56.23
MAO coating	−0.34	21.38
MAO coating with silane sol sealing pores	−0.31	7.76
Corrosion by 1 mol/L HCl solution for 1 h	−0.31	20.42

**Table 3:** The numerical data table of the equivalent circuit.

Samples	R_s_/Ω	C_p_/Ω	n_p_	R_p_/Ω	C_b_/Ω	n_b_	R_b_/Ω
Al substrate	1.5 × 10^−7^	0.8043	0.9554	254.1	-	0.9659	-
MAO coating	1.404 × 10^−7^	0.8074	0.9825	3140	1	0.9784	164,020
Sealed pores	0.214 × 10^−7^	0.6856	0.9631	189,200	1	0.9558	364,300
Corroded coating	3.128 × 10^−7^	0.937	0.9621	54,100	0.5852	0.9687	206,500

## Data Availability

The data presented in this study are available upon request from the corresponding author.
